# Supplementary material for: Towards successful business process improvement – An extension of change acceleration process model
Source: PLoS One. 2019 Nov 27;14(11):e0225669. doi: 10.1371/journal.pone.0225669 (PMC6881029; doi:10.1371/journal.pone.0225669)
Supplement: S1 Appendix — (DOCX) [file pone.0225669.s001.docx]

**APPENDIX-A**

**List of critical factors from the literature along with references**

| CSF Name | Description | Literature Reference |
| --- | --- | --- |
| Involvement of organizations stake holders and leadership | All the organization stakeholders must be involved in the process of BPI as they define the areas in which the organizations need to excel | [84, 17, 85, 18, 19, 20, 24,27, 28,29, 30, 31,33, 34, 35, 36, 86, 87, 88, 89]. |
| Understanding of the process | Complete understanding of the process must established with all the stakeholders | [18 , 21, 22, 28 , 29 , 30, 35, 86]. |
| Performance measurement | Performance management system must be engaged in order to monitor the improvements brought upon by BPI | [21, 23, 24, 27, 28, 30,33,87, 89,90]. |
| Process Improvement road map | The complete action plan must be created and shared with the stakeholders | [24, 28 , 29, 30,31, 33, 34, 91]. |
| Supporting organizational structure | The organization must establish a business process structure that maintains the life (efficiency and effectiveness) of its processes. | [84, 22, 23, 24, 28 29, 30, 31, 33,35, 34, 86, 20] |
| Application of BPI toolbox | The application of a BPI toolkit having all the tools and techniques necessary to execute the BPI project. | [21, 22, 28 , 30, 35, 36, 91]. |
| BPM experienced business process manager | The project manager *must* have significant skills with regard to people change management and stakeholder management. | [17, 19, 22, 23, 24, 27, 28 29, 30, 32,35, 36,86, 93]. |
| Linkage to organization strategy | Sometimes certain tactical processes; proposed as solution to some short term problems, become so closely ingrained in the fabric of an organizations processes that they needed to be sorted out and eliminated. | **[**16**,** 21, 23, 27, 29, 30, 34, 35,86] |
| People change management | Processes are executed either by people, or by people supported by technology. | [84, 17, 23, 21, 22, 24,27, 28, 29, 30, 33, 35, 86, 93]. |
| Learning Organizational Culture | The culture of the organization encourages a learning and support a learning environment. | [84, 25, 27, 30, 31,32, 34, 35, 92] |
| People training and empowerment | People should be provided with an environment in which to work that allows for their creativity and flexibility to perform, provided they have been set and understand their role, goals and targets. | [17, 85, 18, 19, 20, 22, 23, 27, 28, 29, 30, 31, 32, 35, 36, 86, 87, 88 93]. |
| Project initiation and Completions | As soon as BPI projects are completed, a post-implementation review must be conducted to ensure that the lessons learned from one project are transferred to subsequent projects. | [17, 21, 27, 28, 29, 30, 32, 33] |
| Realizing Value | Always let all stakeholders know of the benefits gained from the implementations of quick wins – a great BPM selling tool. | [19, 21, 24,27, 29, 94]. |
| Level of IT investment | A proper level of IT investment is contingent on company’s strategy, other organizational resources, which interact with IT and on the external environment. | [23, 27, 30, 33, 35, 36, 87] |
| Standardization of processes | Standardization ensures that processes are executed in a way that is consistent with specifications and rules. Only the standardized processes bring standardized tasks that can be supported by a proper technological solution. | [21, 22, 23 88, 91] |
| Automation | Refers to the flexibility and use of IT to assist or replace employees in the performance of a business process | [22, 23, 27, 30, 88, 94**]** |
| Appointment of process owners | All processes should have a clearly defined owner who reviews process performance and is responsible for its continuous improvement. In such way process owners are in charge of assuring the dynamic improvement of the capabilities of business processes. | [20, 21, 23, 27, 28, 30, 31, 92, 88] |
| Scope change management | Allow changes to scope only through a mature scope change control process | [85, 21, 28, 30, 31, 32, 92, 86]. |
| Communication | Communication at with all stakeholders including the end users | [20, 22, 27, 28, 30, 31, 34, 35, 92, 86]. |
| Use of external support and expertise | Use of consultants for BPI | [20, 28, 31,.37] |
| Resources allocation | Ensuring the availability of resources for BPI project | [84, 20, 24, 27, 30, 31, 36, 92, 93] |
| Customer focus | The BPI improvement initiative is focused on the customer. | [28, 30,87, 88] |
