# Supplementary material for: Towards successful business process improvement – An extension of change acceleration process model
Source: PLoS One. 2019 Nov 27;14(11):e0225669. doi: 10.1371/journal.pone.0225669 (PMC6881029; doi:10.1371/journal.pone.0225669)
Supplement: S1 Questionnaires — (DOCX) [file pone.0225669.s004.docx]

**Selection Of Most Critical Success Factors**

### ROUND 1

Please provide your answer by using the tick (🗸) mark.

| 1. Name: |  |
| --- | --- |
| 1. Organization Name: |  |
| 1. Your Position in the Organization |  |
| 1. Gender | □ Male □ Female |
| 1. Your Experience in Business Process Improvement | □ Less than 1 Year □ 1-3 Years  □ 4-6 Years □ More than 6 years |
| 1. Your Educational Level | □ Intermediate □ Graduation  □ Master □ MPhil/PhD |

| **Critical Success Factors** | **Do You Consider this to be a CSF?** | |
| --- | --- | --- |
|  | **Yes** | **No** |
| **Organizations stake holders and leadership** All the organization stakeholders must be involved in the process of BPI as they define the areas in which the organizations need to excel |  |  |
| **Understanding of the process** Complete understanding of the process must established with all the stakeholders |  |  |
| **Performance measurement** Performance management system must be engaged in order to monitor the improvements brought upon by BPI |  |  |
| **Process Improvement Road Map** The complete action plan must be created and shared with the stakeholders |  |  |
| **Supporting organizational structure** The organization must establish a business process structure that maintains the life (efficiency and effectiveness) of its processes. |  |  |
| **BPI toolbox** BPI toolkit has all the tools and techniques necessary to execute the BPI project. |  |  |
| **BPM experienced business process manager** The project manager *must* have significant skills with regard to people change management and stakeholder management. |  |  |
| **Linkage to organization strategy** The Improvement initiative under consideration must stay aligned with the organizational strategy. |  |  |
| **People change management** Processes are executed either by people, or by people supported by technology. So, People change management skills and techniques should be present. |  |  |
| **People training and empowerment** People should be provided with an environment to work that allows for their creativity and flexibility to perform, provided they have been set and understand their role, goals and targets. |  |  |
| **Project Initiation and Completions** As soon as BPI projects are completed, a post-implementation review must be conducted to ensure that the lessons learned from one project are transferred to subsequent projects. |  |  |
| **Realizing Value** Always let everyone (all stakeholders) know of the benefits gained from the implementations of quick wins – a great BPM selling tool |  |  |
| **Level of IT investment** A proper level of IT investment is contingent on company’s strategy, other organizational resources, which interact with IT and on the external environment. |  |  |
| **Standardization of processes** Standardization ensures that processes are executed in a way that is consistent with specifications and rules. Only the standardized processes bring standardized tasks that can be supported by a proper technological solution. |  |  |
| **Automation** Refers to the flexibility and use of IT to assist or replace employees in the performance of a business process. |  |  |
| **Appointment of process owners** All processes should have a clearly defined owner who reviews process performance and is responsible for its continuous improvement. In such way process owners are in charge of assuring the dynamic improvement of the capabilities of business processes. |  |  |
| **Informatization** Balance of information given to people handling the process. Information overload and scarcity can both be a challenge. |  |  |
| **Adequacy of "Risk Management"** Adequacy of company-wide education on the concepts of risk management, Maturity of an organization's processes for assigning ownership of risks, Adequacy with which a visible risk register is maintained, Adequacy of an up-to-date risk management plan |  |  |
| **Restricting life of project** Keep project (or project stage duration) as far below 3 years as possible (1 year is better) |  |  |
| **Scope change management** Allow changes to scope only through a mature scope change control process |  |  |
| **Communication** Communication with all stakeholders including the end users |  |  |
| **Problem difficulty** Describes the impact and scope of the process to be improved |  |  |
| **Use of external support and expertise** Use of consultants for BPI |  |  |
| **Resources allocation** Ensuring the availability of resources for BPI project |  |  |
| **Customer focus**  Focus of the improvement and the changes it imposes on the customer. |  |  |

**Any other Critical Success Factors you would like to Add in the list, Feel free to enlist your Valued opinion:**

________________________________________________________________________________________________________________________________________________________________________________________________________________________________________________________________**THANKS FOR YOUR COOPERATION**

Many, many Sincere thanks for your assistance and cooperation in completing this questionnaire

**Selection Of Most Critical Success Factors**

### ROUND 2

Please provide your answer by using the tick (🗸) mark.

| 1. Name: |  |
| --- | --- |
| 1. Organization Name: |  |
| 1. Your Position in the Organization |  |
| 1. Gender | □ Male □ Female |
| 1. Your Experience in Business Process Improvement | □ Less than 1 Year □ 1-3 Years  □ 4-6 Years □ More than 6 years |
| 1. Your Educational Level | □ Intermediate □ Graduation  □ Master □ MPhil/PhD |

| **Critical Success Factors** | **Strongly Agree**  **(5)** | **Agree**  **(4)** | **Neutral**  **(3)** | **Disagree**  **(2)** | **Strongly Disagree**  **(1)** |
| --- | --- | --- | --- | --- | --- |
| **Organizations stake holders and leadership** All the organization stakeholders must be involved in the process of BPI as they define the areas in which the organizations need to excel |  |  |  |  |  |
| **Understanding of the process** Complete understanding of the process must established with all the stakeholders |  |  |  |  |  |
| **Performance measurement** Performance management system must be engaged in order to monitor the improvements brought upon by BPI |  |  |  |  |  |
| **Process Improvement Road Map** The complete action plan must be created and shared with the stakeholders |  |  |  |  |  |
| **Supporting organizational structure** The organization must establish a business process structure that maintains the life (efficiency and effectiveness) of its processes. |  |  |  |  |  |
| **BPM experienced business process manager** The project manager *must* have significant skills with regard to people change management and stakeholder management. |  |  |  |  |  |
| **Linkage to organization strategy** The Improvement initiative under consideration must stay aligned with the organizational strategy. |  |  |  |  |  |
| **People change management** Processes are executed either by people, or by people supported by technology. So, People change management skills and techniques should be present. |  |  |  |  |  |
| **People training and empowerment** People should be provided with an environment in which to work that allows for their creativity and flexibility to perform, provided they have been set and understand their role, goals and targets. |  |  |  |  |  |
| **Realizing Value** Always let everyone (all stakeholders) know of the benefits gained from the implementations of quick wins – a great BPM selling tool |  |  |  |  |  |
| **Appointment of process owners** All processes should have a clearly defined owner who reviews process performance and is responsible for its continuous improvement. In such way process owners are in charge of assuring the dynamic improvement of the capabilities of business processes. |  |  |  |  |  |
| **Adequacy of "Risk Management"** Adequacy of company-wide education on the concepts of risk management, Maturity of an organization's processes for assigning ownership of risks, Adequacy with which a visible risk register is maintained, Adequacy of an up-to-date risk management plan |  |  |  |  |  |
| **Scope change management** Allow changes to scope only through a mature scope change control process |  |  |  |  |  |
| **Communication** Communication with all stakeholders including the end users |  |  |  |  |  |
| **Resources allocation** Ensuring the availability of resources for BPI project |  |  |  |  |  |
| **Customer focus**  Focus of the improvement and the changes it imposes on the customer. |  |  |  |  |  |

**THANKS FOR YOUR COOPERATION**

Many, many Sincere thanks for your assistance and cooperation in completing this questionnaire

**Selection Of Most Critical Success Factors**

### ROUND 3

Please provide your answer by using the tick (🗸) mark.

| 1. Name: |  |
| --- | --- |
| 1. Organization Name: |  |

| **Critical Success Factors** | **PANEL RATING** | | **Your Previous rating** | **Your New Rating** |
| --- | --- | --- | --- | --- |
|  | **Mean** | **SD** |  |  |
| **Organizations stake holders and leadership** All the organization stakeholders must be involved in the process of BPI as they define the areas in which the organizations need to excel |  |  |  |  |
| **Understanding of the process** Complete understanding of the process must established with all the stakeholders |  |  |  |  |
| **Performance measurement** Performance management system must be engaged in order to monitor the improvements brought upon by BPI |  |  |  |  |
| **Process Improvement Road Map** The complete action plan must be created and shared with the stakeholders |  |  |  |  |
| **Supporting organizational structure** The organization must establish a business process structure that maintains the life (efficiency and effectiveness) of its processes. |  |  |  |  |
| **People change management** Processes are executed either by people, or by people supported by technology. So, People change management skills and techniques should be present. |  |  |  |  |
| **People training and empowerment** People should be provided with an environment in which to work that allows for their creativity and flexibility to perform, provided they have been set and understand their role, goals and targets. |  |  |  |  |
| **Realizing Value** Always let everyone (all stakeholders) know of the benefits gained from the implementations of quick wins – a great BPM selling tool |  |  |  |  |
| **Appointment of process owners** All processes should have a clearly defined owner who reviews process performance and is responsible for its continuous improvement. In such way process owners are in charge of assuring the dynamic improvement of the capabilities of business processes. |  |  |  |  |
| **Scope change management** Allow changes to scope only through a mature scope change control process |  |  |  |  |
| **Communication** Communication with all stakeholders including the end users |  |  |  |  |
| **Resources allocation** Ensuring the availability of resources for BPI project |  |  |  |  |
| **Customer focus**  Focus of the improvement and the changes it imposes on the customer. |  |  |  |  |

**THANKS FOR YOUR COOPERATION**

EFA Questionnaire

*Required

1. **Name *** 2. **Organization Name *** 3. **Position In Organization ***

4. **Gender *** *Mark only one oval.* Female Male 5. **Education:**

6. **Experience *** *Mark only one oval.*

Less than one year 1-3 Years 4-6 Years More than 6 Years

"The respondent is requested to think of a recent BPI project and answer all questions with respect to that specific project."

| **Factors** | 1 | 2 | 3 | 4 | 5 |
| --- | --- | --- | --- | --- | --- |
| 1. **Communication *** | | | | | |
| Project team members engaged in open and honest communication. |  |  |  |  |  |
| The quality of project communication was poor. |  |  |  |  |  |
| Project heavily relied on verbal communication. |  |  |  |  |  |
| Project heavily relied upon formal written communication. |  |  |  |  |  |
| 2. **Realizing Value *** | | | | | |
| The outcome of the BPI project added value to the business operations. |  |  |  |  |  |
| Compared to other projects at the firm, this project was of high value |  |  |  |  |  |
| 3. **Understanding of the Process *** | | | | | |
| Team members had a common understanding of the technologies used in the development process. |  |  |  |  |  |
| Workers had a deeper understanding of our firm and customers. |  |  |  |  |  |
| The project executive had a solid understanding of the systems development process. |  |  |  |  |  |
| There was a poor understanding of key process issues of BPI project. |  |  |  |  |  |
| 4. **Resource Allocation *** | | | | | |
| Sufficient decision authority existed regarding resource allocation for BPI project by BPM manager |  |  |  |  |  |
| Team members participated in decisions regarding resource allocation of BPI project. |  |  |  |  |  |
| All project members agreed to the commitment of their time as per the project plan. |  |  |  |  |  |
| 5. **Organization Leadership and Stakeholders *** | | | | | |
| There was commitment and support from the top management during the BPI implementation process |  |  |  |  |  |
| Leadership was effective and creative in taking decisions. |  |  |  |  |  |
| 6. **Involvement of Process Owners *** | | | | | |
| BPI project included process owners throughout the BPI effort |  |  |  |  |  |
| BPI project identified process owners who were responsible for the entire business process. |  |  |  |  |  |
| 7. **People Change Management *** | | | | | |
| Any change or modification in BPI project was ethically guided. |  |  |  |  |  |
| The change management support was available whenever needed. |  |  |  |  |  |
| The change management consultants understood problems well.  The change management consultants resolved the problems. |  |  |  |  |  |
| Changes suggested by management were well informed and had valid reasons |  |  |  |  |  |
| 8 **Performance Management *** | | | | | |
| Adequate system of reward or punishment based on rigorous management of BPI project members performance existed in project. |  |  |  |  |  |
| Staff were allowed to set their goals, monitor their own performance, in relation to their work targets. |  |  |  |  |  |
| 9 **Supporting Organizational Structure *** | | | | | |
| Application of the BPI project rapidly changed the organization structure |  |  |  |  |  |
| The hierarchical relationship of the BPM/BPI function to the overall organizational structure was adequate. |  |  |  |  |  |
| Different organization structures were integrated as a result of the BPI project implementation. |  |  |  |  |  |
| **Please Specify Organizational Structure *** (Projectized/Matrix etc) |  | | | | |
| 10 **People Training and Empowerment *** | | | | | |
| Training required to perform the BPI project was readily available in manuals. |  |  |  |  |  |
| There was difficulty in providing training to employees in the skills required for this project. |  |  |  |  |  |
| Training people to deal with the BPI project involved substantial commitments of time and money. |  |  |  |  |  |
| 11. **Scope Change Management *** | | | | | |
| The planning team briefed senior management about the scope of the BPI project |  |  |  |  |  |
| The scope of the project was clearly defined. |  |  |  |  |  |
| 12. **Customer Focus *** | | | | | |
| The BPI intervention really focused on customer. |  |  |  |  |  |
| BPI project contributed significantly to establishing strong and continuous relationship with customers |  |  |  |  |  |
| Customers’ complaints, lost customer analysis and feedback was used to improve the product/ service |  |  |  |  |  |
| 13. **Process Improvement Road Map *** | | | | | |
| The project plan and estimates were realistic. |  |  |  |  |  |
| Corrective action was taken proactively when actual results deviated from the project plan. |  |  |  |  |  |
| The project’s actual results on the project plan were compared regularly with estimates in the project plan. |  |  |  |  |  |
| BPI intervention quality was assessed and compared to the quality goals in the project plan. |  |  |  |  |  |

**Thanks for your cooperation**
