# Supplementary material for: Towards successful business process improvement – An extension of change acceleration process model
Source: PLoS One. 2019 Nov 27;14(11):e0225669. doi: 10.1371/journal.pone.0225669 (PMC6881029; doi:10.1371/journal.pone.0225669)
Supplement: Attachment — Submitted filename: PLOS ONE reviewer report PONE-D-19-20062.pdf [file pone.0225669.s005.pdf]

PLOS ONE reviewer report

PONE-D-19-20062

Paper: *Ensuring Business Process Improvement Success- An Extension of Change Acceleration Process Model*

Report

This is an interesting paper covering the topic of business management/change management specifically in the area of business process improvement. Given that businesses are ever competitive and dynamic, companies need to constantly upgrade and evolve both in terms of their products and their operations – this is more pronounced for some sectors. As such, the sector chosen in the paper is certainly relevant as the telecommunication industry is highly competitive with an ever changing business landscape coupled with constant technological changes taking place.

Overall, I think this paper is fairly well-written and executed with reliable methodology. It also delivers some interesting results that may prove useful for the relevant stakeholders.

That said, I do have some comments about the paper and they are as follows:

- The introduction section (on business, change management etc.. in general) should a little longer before the paper jumps into *BPI* materials.
- A section (or sub-section) devoted to *BPI* after the introduction section would improve the flow of the paper and also provide some general and basic insights to the use of BPI – this would be especially useful (easier) for general readers (non-specialists in the area).
- Since there are many use of acronyms, the authors would be well-advised to provide the full term when the item appears for the first time – e.g., in page 2 first line of the second paragraph, what does GE stands for? Also, at times, the authors seem to use the full term and its acronyms interchangeably at different points of the paper (e.g., CSF was already in use in 1 but appears in full in page 4).
- A dedicated (can also be brief if needed) section on the *Pakistan telecommunication sector* would also be good for readers to better appreciate the topic – just to ease readers into the topic with some knowledge of the sector, the number of players and perhaps some industry statistics as well. At the very least to provide some context as to why changes in organization may be crucial in such dynamic sector thus warrants the use (continued use) of BPI. At the moment, the paper feels a bit too skewed towards the business process discussions (i.e., BPI, CAP etc..) – I think there a need to view the topic also from a general business environment sense thus some re-

organisations and additions would greatly improve the appeal of the paper in my opinion, especially for the more casual readers.

- The section on *Change Accelerated Process Analysis* should come after the proposed section on BPI and not after the Literature Review – this to improve the flow of the paper.
- The literature review needs to be more critical and better informed – a little too brief given that it is less than a page long.
- The last paragraph in page 3 should not be part of the literature review – move this to the section on *Research Design*.
- While the paper is fairly well-written, it is not without its typos/grammatical issues and they are quite a few throughout the paper. Also, some parts appears to be a little sloppy (e.g., the word Figure does not need to be bolded, some in-text referencing and reporting of the sources of the figures [in the case of Fig 4 in page 4] not correct, etc..), spacing in between paragraphs a little neglected, etc… so some editorial improvements are necessary. In fact, I think it would be good to have the paper proofread (if possible) before further submission(s).
- In page 6, the number of respondents was 26 and 22 in the two rounds respectively – are these sufficient? The authors claimed that a previous literature indicated that the size may vary from 4 to 3000 --- does this suggests that the number of respondents in this paper is on the low side? Just a suggestion, perhaps to cite a previous work that has a sample size around this amount (e.g., anything around 30 respondents or less)?
- Some brief background of the respondents may be good – e.g., age, position in organization. Ideally, these managers should be similar in terms of their job designation/title/position otherwise the responses collected may suffer from some *position bias*? If they differed markedly or somewhat markedly, the authors may want to offer some explanation as to why this is not a problem or at the very least, not critical.
- The authors should provide all the questionnaires in the Appendix (e.g., the Delphi questionnaires, the EFA questionnaire etc..).
- Did the authors consider adding any new CSFs? Why not? It seems that all the CSFs in the exhaustive list were sourced from other literatures. Did the questionnaire design involve any open-ended sections to elicit new information (potentially new CSF?)? Just curious if the authors have thought of this --- new CSFs would certainly be an additional if not good contribution.
- In page 7 (under *Round 2 Ranking, Results & Analysis*), the respondents were divided into 2 groups, i.e., 'Leadership' and 'Management'. How did the authors decide on the allocation of which respondent to which group? Also, how many were placed under Leadership and how many, Management?
- In page 8 (under *Round 3 Ranking, Results & Analysis*), how many respondents were there?
- Where's Appendix B?
- Some sort of alignment issue in column 1 in Table IV?

- In page 18 – perhaps a bit more explanation on why (and what does it mean) and the implications of *customer focus* having a negative factor loading.

Overall, the paper's contribution is presented very clearly in the paper, i.e., presenting an extension of CAP for the BPI projects specifically in the telecommunication industry. While this finding was derived through a fairly thorough methodology (I have no issues with it), I wonder if the results (i.e., the proposed extended model) is a little general – this model seem as though it could be applied to any industry in my opinion. I am not sure if this is a concern but perhaps the authors can offer a more convincing discussion as to why the proposed extended model is so crucial, specifically to the telco industry – the explanation in the last paragraph of page 18 and first paragraph in page 19 offers very general discussions on the model and its applications. While this fine but the paper does make a strong assertion both at the onset and throughout the paper that it (the research exercise) is specific to the telco industry so one may expect the discussions to be more industry specific or at the very least offer some insights as to how the model would contribute to making BPI projects more successful in the context of the telco sector. Perhaps one way to show this would be to discuss how the proposed model is different from others (e.g., previous models that were derived from research using respondents from other sectors/industries)?
