# Supplementary material for: Towards successful business process improvement – An extension of change acceleration process model
Source: PLoS One. 2019 Nov 27;14(11):e0225669. doi: 10.1371/journal.pone.0225669 (PMC6881029; doi:10.1371/journal.pone.0225669)
Supplement: Attachment — Submitted filename: Response to Reviewers.docx [file pone.0225669.s006.docx]

Dear Editor,

PLOS One,

October 3, 2019

Guest Editors,

PLOS One,

Dear Guest Editors,

Thank you for your letter and the opportunity to revise our manuscript **ID: PONE-D-19-20062-** "**Ensuring Business Process Improvement Success- An Extension of Change Acceleration Process Model**”. *(Please note that the paper title has been revised to accommodate reviewer's comments).*

Please assign this manuscript to the **same reviewers** who have reviewed and raised their concerns earlier for this manuscript **ID: PONE-D-19-20062**

The suggestions offered by the reviewers have been immensely helpful, and we also appreciate your insightful comments on revising **the title** and other **parts** of the paper. I have included the reviewer comments immediately after this letter and responded to them individually, indicating exactly how we addressed each concern or problem and describing the changes we have made. The revisions have been approved by all four authors and I have again been chosen as the corresponding author. I have moved Aamer Hanif to second author position based upon his contribution to the paper.

We would also like to thank the reviewers for a thorough review and useful suggestions that enabled the manuscript to be improved. Most of the revisions prompted by the reviewers’ comments are minor and require no further explanation than what appears in my responses below.

Regarding more minor matters, we have now changed our phrasing of the abstract, introduction and discussion section. We hope the revised manuscript will better suit the PLOS ONE Journal and are happy to consider further revisions, and we thank you for your continued interest in our research. We are uploading (a) our point-by-point response to the comments (below) (response to reviewers), (b) a revised with track changes c) a clean updated manuscript and (d) supporting data files.

**Please address all correspondence concerning this manuscript to me by e-mail at: *maha.syed@gmail.com*.**

Sincerely,

Maha Syed,

Corresponding Author.

Response to reviewers

| Concern1 | Please ensure that your manuscript meets PLOS ONE's style requirements, including those for file naming |
| --- | --- |
| Response | The authors have updated the style requirements along with file names |
|  |  |
| Concern2 | Please include additional information regarding the survey or questionnaire used in the study and ensure that you have provided sufficient details that others could replicate the analyses |
| Response | The survey questionnaires have been provided as supporting files |
|  |  |
| Concern3 | The data should be provided as part of the manuscript or its supporting information, or deposited to a public repository. |
| Response | The data files have been provided in supporting files |
|  |  |
| Concern4 | While revising your submission, please upload your figure files to the Preflight Analysis and Conversion Engine (PACE) digital diagnostic tool, <https://pacev2.apexcovantage.com/>. PACE helps ensure that figures meet PLOS requirements. |
| Response | Figure requirements have been checked. |
|  |  |
| Concern5 | A dedicated (can also be brief if needed) section on the Pakistan telecommunication sector would also be good for readers to better appreciate the topic |
| Response | The required section has been included in the manuscript in Page 2 as a subheading for introduction. |
|  |  |
| Concern6 | Some brief background of the respondents may be good – e.g., age, position in organization. Ideally, these managers should be similar in terms of their job designation/title/position otherwise the responses collected may suffer from some position bias? |
| Response | The brief background and demographics have been added under the sub-heading sample for both Delphi and EFA |
| Concern7 | Did the authors consider adding any new CSFs? Why not? It seems that all the CSFs in the exhaustive list were sourced from other literatures. Did the questionnaire design involve any open-ended sections to elicit new information (potentially new CSF?)? |
| Response | Delphi analysis invites participants to include any CSF they deem significant, in Round 1. in this case no additional CSF was suggested by the Panel. |
|  |  |
| Concern8 | Research design is prone to biases. |
| Response | This point has been addresses in the section on “sampling and use of experts of Delphi” with a reference to literature. |
|  |  |
| Concern9 | Based on the analysis, stating that the proposed model "ensure" BPI success is an extreme statement. |
| Response | The specified statement has been changed and also the paper title has been modified to address this extreme statement. |
|  |  |
| Concern10 | Outdated references must be updated. |
| Response | The authors have updated the references and some new references have been added. |
|  |  |
| Concern11 | Where’s Appendix B? |
| Response | Reference to Appendix B was made by mistake and has been fixed. |
|  |  |
| Concern12 | Some sort of alignment issue in column 1 in Table IV? |
| Response | All the tables have been re-aligned and updated |
|  |  |
| Concern13 | Figures used are not clear. (instead of copy pasting images from the web, i suggest to duplicate it) |
| Response | All figures have been recreated. |
|  |  |
| Concern14 | The authors should provide all the questionnaires in the Appendix (e.g., the Delphi questionnaires, the EFA questionnaire etc..). |
| Response | The questionnaires have been added in Appendix B |
|  |  |
| Concern15 | In page 18 – perhaps a bit more explanation on why (and what does it mean) and the implications of customer focus having a negative factor loading |
| Response | The implication of negative factor loading has been discussed in the paper by explaining it in the discussion section of the paper. |
|  |  |
| Concern16 | Since there are many use of acronyms, the authors would be well-advised to provide the full term when the item appears for the first time – e.g., in page 2 first line of the second paragraph, what does GE stands for? |
| Response | GE stands for General Electric. All the acronyms have been updated. |
|  |  |
| Concern17 | While the paper is fairly well-written, it is not without its typos/grammatical issues and they are quite a few throughout the paper. Also, some parts appears to be a little sloppy (e.g., the word Figure does not need to be bolded, some in-text referencing and reporting of the sources of the figures [in the case of Fig 4 in page 4] not correct, etc..), spacing in between paragraphs a little neglected, etc… so some editorial improvements are necessary. In fact, I think it would be good to have the paper proofread (if possible) before further submission(s). |
| Response | The paper has been proofread and updated accordingly. |
|  |  |
| Concern18 | I am not sure if this is a concern but perhaps the authors can offer a more convincing discussion as to why the proposed extended model is so crucial, specifically to the telco industry – the explanation in the last paragraph of page 18 and first paragraph in page 19 offers very general discussions on the model and its applications. |
| Response | The discussion has been improved to be clearer and convincing. A subsection “Utility of extended model” has been added to the paper. |
|  |  |
| Concern19 | One may expect the discussions to be more industry specific or at the very least offer some insights as to how the model would contribute to making BPI projects more successful in the context of the telco sector. |
| Response | The utility of extended model in telecom sector has been highlighted by adding an exclusive sub-section in the paper. |
| Concern20 | The novelty of this work is not clear. (Must be address in the discussion section) |
| Response | A subsection explaining the novelty of this work has been added. |
|  |  |
| Concern21 | The extended CAP model is not clearly defined. How is the proposed model better than existing methods? |
| Response | The rationale behind extended model is explained in subsection “Extension of CAP model” |
|  |  |
| Concern22 | In page 7 (under Round 2 Ranking, Results & Analysis), the respondents were divided into 2 groups, i.e., 'Leadership' and 'Management'. How did the authors decide on the allocation of which respondent to which group? Also, how many were placed under Leadership and how many, Management? |
| Response | Director level and above have been grouped together as Leadership.  Managers and senior managers have been grouped as 'Management' |
|  |  |
| Concern23 | In page 8 (under Round 3 Ranking, Results & Analysis), how many respondents were there? |
| Response | 22 respondents, also reflected in the paper. |
|  |  |
| Concern24 | In page 6, the number of respondents was 26 and 22 in the two rounds respectively – are these sufficient? The authors claimed that a previous literature indicated that the size may vary from 4 to 3000 --- does this suggests that the number of respondents in this paper is on the low side? Just a suggestion, perhaps to cite a previous work that has a sample size around this amount (e.g., anything around 30 respondents or less)? |
| Response | Yes the number of respondents is 22 which is applicable in this case. Respondents less than 15 have also been used in similar studies. Same has been reflected in the paper. |
|  |  |
| Concern25 | Introduction and Literature review need to be improved. The flow and structure of the paper is not well arranged. |
| Response | The manuscript has been revised and updated with improved structure. |
|  |  |
| Concern26 | A section (or sub-section) devoted to BPI after the introduction section would improve the flow of the paper and also provide some general and basic insights to the use of BPI – this would be especially useful (easier) for general readers (non-specialists in the area). |
| Response | The manuscript has been revised and a few lines devoted to BPI have been added |
|  |  |
| Concern27 | The section on Change Accelerated Process Analysis should come after the proposed section on BPI and not after the Literature Review – this to improve the flow of the paper. |
| Response | The proposed changes have been made |
|  |  |
| Concern28 | The literature review needs to be more critical and better informed – a little too brief given that it is less than a page long. |
| Response | The literature review has been revised |
|  |  |
| Concern29 | The last paragraph in page 3 should not be part of the literature review – move this to the section on Research Design. |
| Response | The proposed changes have been made in the paper. |
